# Suicide and Microglia: Recent Findings and Future Perspectives Based on Human Studies

**DOI:** 10.3389/fncel.2019.00031

**Published:** 2019-02-13

**Authors:** Hisaomi Suzuki, Masahiro Ohgidani, Nobuki Kuwano, Fabrice Chrétien, Geoffroy Lorin de la Grandmaison, Mitsumoto Onaya, Itaru Tominaga, Daiki Setoyama, Dongchon Kang, Masaru Mimura, Shigenobu Kanba, Takahiro A. Kato

**Affiliations:** ^1^National Hospital Organization Shimofusa Psychiatric Medical Center, Chiba, Japan; ^2^Department of Neuropsychiatry, Graduate School of Medical Sciences, Kyushu University, Fukuoka, Japan; ^3^Neuropathology Department, Sainte-Anne Hospital, Paris, France; ^4^Human Histopathology and Animal Models Laboratory, Institute Pasteur, Paris, France; ^5^Department of Pathology and Forensic Medicine, Raymond Poincaré University Hospital, Garches, France; ^6^Department of Clinical Chemistry and Laboratory Medicine, Graduate School of Medical Sciences, Kyushu University, Fukuoka, Japan; ^7^Department of Neuropsychiatry, Keio University School of Medicine, Tokyo, Japan

**Keywords:** suicide, microglia, neuroinflammation, depression, postmortem, PET imaging, tryptophan-kynurenine pathway, iMG cells

## Abstract

Suicide is one of the most disastrous outcomes for psychiatric disorders. Recent advances in biological psychiatry have suggested a positive relationship between some specific brain abnormalities and specific symptoms in psychiatric disorders whose organic bases were previously completely unknown. Microglia, immune cells in the brain, are regarded to play crucial roles in brain inflammation by releasing inflammatory mediators and are suggested to contribute to various psychiatric disorders such as depression and schizophrenia. Recently, activated microglia have been suggested to be one of the possible contributing cells to suicide and suicidal behaviors *via* various mechanisms especially including the tryptophan-kynurenine pathway. Animal model research focusing on psychiatric disorders has a long history, however, there are only limited animal models that can properly express psychiatric symptoms. In particular, to our knowledge, animal models of human suicidal behaviors have not been established. Suicide is believed to be limited to humans, therefore human subjects should be the targets of research despite various ethical and technical limitations. From this perspective, we introduce human biological studies focusing on suicide and microglia. We first present neuropathological studies using the human postmortem brain of suicide victims. Second, we show recent findings based on positron emission tomography (PET) imaging and peripheral blood biomarker analysis on living subjects with suicidal ideation and/or suicide-related behaviors especially focusing on the tryptophan-kynurenine pathway. Finally, we propose future perspectives and tasks to clarify the role of microglia in suicide using multi-dimensional analytical methods focusing on human subjects with suicidal ideation, suicide-related behaviors and suicide victims.

## Introduction

Suicide is one of the most serious global mental health issues, and close to 900,000 people die due to suicide every year (Chesney et al., 2014). Suicide occurs throughout the human lifespan and is the second leading cause of death among persons 15–29 years old (WHO, 2018). Effective interventions are critically needed to prevent suicide (Mann et al., 2005; Nakagami et al., 2018). Psychosocial factors such unemployment, poverty, family conflicts and health issues are widely known to be causes of suicide (Rubenowitz et al., 2001).

Moreover, suicide is one of the most disastrous outcomes during psychiatric clinical practice (Thornicroft and Sartorius, 1993). Suicidal ideation, suicidal attempts and other suicide-related behaviors occur especially during the course of depression, and suicide is also prevalent in many other psychiatric disorders such as alcohol addiction, personality disorder and schizophrenia (Chesney et al., 2014). Recently, biological abnormalities underlying such psychiatric disorders have been revealed especially based on animal model studies and human brain studies such as postmortem studies and brain imaging studies with living patients. Recent advances in biological psychiatry have suggested a positive relationship between some specific brain abnormalities and specific symptoms in schizophrenia, depression and other psychiatric disorders whose organic bases were previously completely unknown (Harrison, 1999; Kuperberg et al., 2003; Nobuhara et al., 2006; Fields, 2008; Penzes et al., 2011). The biological factors of suicide have not been well clarified, while some biological basis may exist. Animal model research focusing on psychiatric disorders has a long history, however, there are only limited animal models that can properly express psychiatric symptoms. In particular, to our knowledge, animal models of human suicidal behaviors have not been established (Gould et al., 2017). Suicide is believed to be limited to humans, therefore human subjects should be the targets of research despite various ethical and technical limitations.

Neuroinflammation is suggested to be linked to suicide (Pandey et al., 2012, 2018). Microglia, immune cells in the brain, are regarded to play crucial roles in neuroinflammation *via* releasing inflammatory mediators and are suggested to contribute to various psychiatric disorders (Monji et al., 2009, 2013; Kato et al., 2011a, 2013b,c; Kato and Kanba, 2013). Recently, activated microglia have been suggested to be possible contributing cells to suicide *via* various mechanisms especially the tryptophan-kynurenine pathway, thus we herein introduce human biological studies focusing on suicide and microglia. We first present recent neuropathological studies using the human postmortem brain of suicide victims. Second, we demonstrate recent findings based on positron emission tomography (PET) imaging and peripheral blood biomarker analysis on living subjects with suicide-related behaviors. Finally, we propose future perspectives and tasks to clarify the role of microglia in suicide using multi-dimensional analytical methods.

## Microglia

Microglia, immune cells in the brain, are regarded to play crucial roles in brain homeostasis and inflammation *via* phagocytosis and/or releasing pro- and anti- inflammatory mediators such as cytokines and chemokines (Block and Hong, 2005). Psychological stress is one of the most frequent triggers of suicide (Hawton and van Heeringen, 2009). Rodent studies have revealed that acute and chronic stress based on social defeat model and restraint model induce microglial activation in various brain regions (Sugama et al., 2007; Tynan et al., 2010; Hinwood et al., 2012; Ohgidani et al., 2016). Human microglia research is difficult to conduct because of difficulty in analysis of microglia in human subjects based on ethical and technical issues (Ohgidani et al., 2015). To our knowledge, human microglia analysis during the course of psychological stress has not been conducted, while our previous pharmacological study with healthy volunteers using minocycline, an antibiotic with suppressing microglial activation in rodents, has indirectly suggested that human social-decision making in stressful situations is unconsciously controlled by microglia (Kato et al., 2012, 2013b; Watabe et al., 2013). Postmortem brain analysis and PET imaging are two major methods to estimate microglial activation in human subjects, and these studies have suggested activation of human microglia in the brain of patients with various psychiatric disorders (Kato et al., 2013b). Here, we introduce human biological studies using these techniques focusing on suicide and microglia.

## Postmortem Neuropathological Studies Focusing on Microglia and Suicide

In 1919, Pio del Rio-Hortega initially characterized morphological phenotypes of microglia and described that ramified microglia transform into amoeboid form in different environments of brain pathology (Sierra et al., 2016). Even today, these findings are considered as the base of microglial biology, and morphological change from ramified to amoeboid shape indicate functional shifts from resting state to active state (Kettenmann et al., 2011).

Here, we introduce the following five original studies using the human postmortem brain of patients with psychiatric disorders including suicide victims. An overview of these publications was summarized in Supplementary Table S1.

Steiner et al. (2006) first suggested the possible link between suicide and microglial activation, analyzing the morphological characteristics of microglia by immunohistochemistry with HLA-DR as a microglial marker in some regions of the brain of psychiatric patients including suicide victims. Cell density of microglia was not significantly different between cases with schizophrenia, depressive state of affective disorder and non-psychiatric control subjects. However, significant microgliosis (i.e., increased microglial density) was observed in the dorsolateral prefrontal cortex (DLPFC), anterior cingulate cortex (ACC) and mediodorsal thalamus (MD) of suicide victims (Steiner et al., 2006, 2008).

Schnieder et al. (2014) reported a postmortem study of psychiatric disorders including schizophrenia and affective disorder; analyzing the microglial morphology and active state by immunohistochemistry with ionized calcium-binding adapter molecule 1 (Iba-1) and cluster of differentiation 68 (CD68), respectively. Microglial density and active state were not different between diagnostic differences. Interestingly, there were significant effects of suicide on density of activated microglia in ventral prefrontal white matter relative to dorsal area. In addition, Iba-1-positive activated microglia were observed within or in contact with blood vessel walls in dorsal prefrontal white matter in the suicide group (Schnieder et al., 2014).

Torres-Platas et al. (2014b) conducted a case-control study with immunohistochemistry and gene expression analysis using postmortem brains between the cases of suicide victims with major depression and control subjects without psychiatric disease who died of physical illnesses. Total densities of Iba-1 stained microglia and IBA1-immunoreactive (IBA-IR) were not significantly different between the cases of suicide victims with depression and control subjects. The ratio of primed over ramified (“resting”) microglia was significantly increased in suicide victims with depression. The proportion of blood vessels surrounded by a high density of macrophages was more than twice higher in suicide victims with depression. Gene expression of IBA1 and MCP-1 was significantly upregulated in suicide victims with depression (Torres-Platas et al., 2014b).

Brisch et al. (2017) showed that only the subgroup of patients with depression who were not suicide victims revealed significantly lower microglial reaction, i.e., a decreased density of HLA-DR positive microglia vs. both suicide victims with depression and control subjects.

In sum, no significant difference of microglial change was observed between psychiatric patients and healthy controls. On the other hand, microglial morphologies were significantly different (increased cell density and metamorphosis) in the brains of suicide victims. These outcomes have suggested a relationship between microglial activation and suicide beyond the diagnostic classification of psychiatric disorders. Therefore, further larger studies including various psychiatric disorders are needed in order to elucidate the relationship between microglia and suicide beyond the psychiatric diagnostic boundaries.

**Figure 1:**
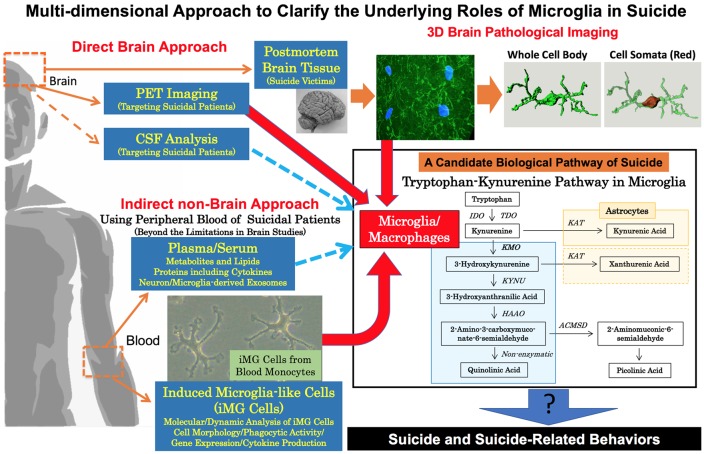
Multi-dimensional approach to clarify the underlying roles of microglia in suicide. To clarify the underlying roles of human microglia in suicide, a multi-dimensional approach should be conducted. Brain neuropathological analysis and PET imaging are both essential in revealing direct pathophysiological evidence of microglia in suicide. Our 3D morphological analysis of microglia in a suicide victim is shown in the upper right. Our method is able to measure the 3D morphological parameter of the whole cell body and cell somata individually (details are shown in Supplementary Document). To overcome the limitations of brain studies in suicide, indirect non-brain approaches are also needed especially to grasp the dynamic roles of microglia in suicide and suicide-related behaviors including suicidal attempts/suicidal ideation. As indirect approaches, we propose the usage of peripheral bloods (plasma/serum and monocytes). Recently, dysregulation of the tryptophan-kynurenine pathway possibly *via* microglial activation has been suggested to be a positive link to suicide and suicide related behaviors. Novel key molecules, especially in the tryptophan/kynurenine pathway, may be discovered by wider analysis of metabolites, lipids and also neuron/microglia-derived exosomes in plasma/serum of suicidal patients. In addition, dynamic analysis of induced microglia-like (iMG) cells from blood monocytes is expected to reveal dynamic and molecular mechanisms of microglia in suicidal behaviors. Finally, deeper mechanisms of microglia in suicide may be discovered by the multi-dimensional combined analysis of both direct and indirect data. ACMSD, amino-β-carboxymuconate-semialdehyde-decarboxylase; HAAO, hydroxyanthranilate 3,4-dioxygenase; IDO, indoleamine 2,3-dioxygenases; KAT, kynurenine aminotransferases; KMO, kynurenine 3-monooxygenases; KYNU, kynureninase; TDO, tryptophan 2,3-dioxygenase; PET, positron emission tomography.

We are presently reconstructing the 3D model of microglia from the human brain of suicide victims with depression by drug overdose (details of sampling information and methods are shown in Supplementary Document). In this 3D model, we have successfully divided the whole cell body and cell somata. Accordingly, we are able to measure 3D morphological parameters such as the surface areas and volumes both of the whole cell body and cell somata individually (in the upper right of Figure 1). Kreisel et al. (2014) have revealed an enlargement of microglial cellular somata in the brains of stress-model mice. Therefore, we believe that our detailed 3D analytical approach can clarify deeper morphological characteristics of microglia in the brain of suicide victims for future investigations.

The above neuropathological studies should be validated by further studies focusing on the following aspects to counter the limitations: (1) larger samples are needed; (2) precision and grouping of subject characteristics such as age, gender, cause of death and postmortem intervals are considered; (3) storage environment including prolonged postmortem interval should be carefully considered. Torres-Platas et al. (2014a) showed that there was no modification of microglia after a post-mortem interval of 43 h before tissue fixation in mice. Similarly, Dibaj et al. (2010) reported that microglial activity and responsiveness remain only for up to 5–10 h post-mortem depending on the premortal condition of the animal. In addition, Eyo and Dailey (2012) showed that nearly half of microglia die within 6 h of sustained oxygen and glucose deprivation. These reports have suggested that postmortem morphological change of microglia may not be significant in human as well. Even though, we should carefully interpret the data of microglia from postmortem brains especially in suicide victims because the time from death by suicide is not precisely calculated; (4) regional specificity should be analyzed; and (5) finally, medication data should be considered as various *in vitro* studies using rodent microglial cells have suggested that some antipsychotics and antidepressants reduce microglial activation by suppressing the release of inflammatory mediators such as inflammatory cytokines and free radicals (Hashioka et al., 2007; Kato et al., 2007, 2008, 2011a,b; Bian et al., 2008; Horikawa et al., 2010; Hashioka, 2011; Müller et al., 2013; Su et al., 2015; Sato-Kasai et al., 2016).

## A Pet Study Focusing on Microglia and Suicide

As shown above, neuropathological studies with postmortem the human brain of suicide victims enable us to reveal activation of microglia based on morphological and histochemical characteristics. However, human postmortem studies especially focusing on suicide victims have limitations because of sampling biases such as methods of death and time period after death. Microglia may continuously be activating even after death, which may influence the pathological conditions of microglia. To resolve this limitation, human living-microglia studies focusing on living persons with suicide risks are of great importance to understand the role of microglia in suicide.

At present, there is no other method than PET imaging that is capable of detecting microglial activation in the brain of human living subjects using ligands of translocator protein (TSPO), also known as a peripheral benzodiazepine receptor (Banati et al., 2000; Rupprecht et al., 2010). Until now, human PET studies using TSPO ligands have suggested over microglial activation in a variety of psychiatric patients such as schizophrenia, at-risk stage of psychosis, autism and depression (van Berckel et al., 2008; Doorduin et al., 2009; Takano et al., 2010; Suzuki et al., 2013; Setiawan et al., 2015, 2018; Bloomfield et al., 2016).

Holmes et al. (2018) have conducted a case-control study using [11C](R)-PK11195 PET to compare TSPO availability in ACC, PFC, and insula between 14 medication-free patients with major depressive disorder (MDD) during a moderate to severe depression severity and 13 matched healthy control subjects. In a *post hoc* analysis, they also compared TSPO availability between patients with and without suicidal thoughts. Multivariate analysis of variance indicated significantly higher TSPO in patients compared with control subjects. The elevation was of large effect size and significant in the ACC. Interestingly, TSPO was not elevated in patients without suicidal thoughts but was significantly increased in those with suicidal thoughts, most robustly in the ACC and insula.

The above PET study has suggested microglial activation during the presence of suicidal ideation in depression. At present, PET imaging is regarded to be the only valid method to evaluate microglial activation in the brain of living human subjects. A variety of activation patterns exist in microglia such as cytokine releases and phagocytosis (Burguillos et al., 2011), and we suspect that some microglial activations may be independent from TSPO pathway and TSPO may detect only limited pathways in microglial activation. Some other ligands targeting microglial activation such as cannabinoid receptor CB2 (CB2R) should be applied for clinical PET studies in psychiatry in addition to TSPO-PET imaging studies (Ni et al., 2018).

## Tryptophan-Kynurenine Pathway on Microglia and Suicide

One of the important biological impacts related to microglial activation is altered regulation of the tryptophan-kynurenine pathway (Dantzer et al., 2011; Myint, 2012). Tryptophan-kynurenine pathway has various neurotoxic metabolites [e.g., 3-hydroxykynurenine (3-HK), quinolinic acid (QUIN), xanthurenic acid (XA)] and neuroprotective metabolites [e.g., kynurenic acid (KA), picolinic acid (PIC), XA; Courtet et al., 2016; Mechawar and Savitz, 2016; Bryleva and Brundin, 2017a, b; Sudol and Mann, 2017; Kanchanatawan et al., 2018; Ogyu et al., 2018]. Interestingly, the synthesis of 3-HK and its downstream metabolites including QUIN mainly takes place in microglia. On the other hand, neuroprotective metabolites are mainly produced by astrocytes. Detailed mechanisms regarding how to control/switch the activation of microglia and astrocytes in the tryptophan-kynurenine pathway have not been well clarified, while some specific enzymes inside microglia and astrocytes have been suggested to play a role (Dantzer et al., 2011; Myint, 2012). Microglia have several enzymes such as kynurenine 3-monooxygenases (KMO) necessary for this pathway, and astrocytes also play important roles in the tryptophan-kynurenine pathway mainly *via* an enzyme, kynurenine aminotransferases (KAT; the lower right part of Figure 1; Dantzer et al., 2011; Myint et al., 2012; Myint, 2012; Mechawar and Savitz, 2016; Bryleva and Brundin, 2017a). In postmortem brain studies of suicide victims (Supplementary Table S2), Steiner et al. (2011) have reported increased expression of microglial QUIN in ACC of suicide victims with depression compared to non-psychiatric control subjects who were not suicide victims. On the other hand, Busse et al. (2015) have shown decreased expression of microglial QUIN in hippocampal regions of suicide victims with depression. Moreover, another postmortem brain study has reported decreased expression not only of QUIN but also indoleamine 2,3-dioxygenase (IDO) 1/2, and tryptophan-2,3-dioxygenase (TDO) in the ventrolateral PFC of suicide victims with depression (Clark et al., 2016). The activation of IDO and TDO, which degrades TRP to KYN along the tryptophan-kynurenine pathway, is suggested to reduce serotonin production through upregulation of tryptophan degradation (Dantzer et al., 2011; Maes et al., 2011; Myint, 2012; Myint et al., 2012; Halaris et al., 2015). Thus, an imbalance of neuroactive metabolites in this pathway *via* microglia may be involved in the biological process of suicide.

In addition to the direct approach using postmortem brains, recent clinical studies using blood and/or cerebrospinal fluid (CSF) have revealed the possible relationship between the tryptophan-kynurenine pathway and suicide (Supplementary Table S2). Sublette et al. (2011) have reported higher levels of plasma TRP and KYN in MDD patients with suicide attempts compared to MDD patients without suicide attempts. Bradley et al. (2015) have shown lower levels of plasma TRP and higher ratio of KYN/TRP (an indicator for the activity of IDO and TDO) in MDD patients with suicide attempts/suicidal ideation compared to HC and MDD patients without suicide attempts/suicidal ideation. Also, Bradley et al. (2015) have shown that KYN/TRP ratio has significantly positive correlation with the severity of suicidal ideation in drug-free MDD patients, suggesting the upregulation of tryptophan-kynurenine pathway in suicidality. On the other hand, using metabolomics, we have recently suggested the downregulation of this pathway (e.g., higher TRP, lower KYN/TRP ratio, KYN, KA, 3-HK, XA) is associated with the severity of suicidal ideation in depressed patients (including non-MDD patients) and/or first-episode drug-naïve MDD patients (Setoyama et al., 2016; Kuwano et al., 2018b). The above contradictory results in blood studies indicate that the regulation of the tryptophan-kynurenine pathway may change according to the time-course of suicide-related behaviors.

In recent longitudinal follow-up studies of suicidal patients, Bay-Richter et al. (2015) have reported higher levels of CSF QUIN and lower levels of CSF KA in psychiatric patients with suicide attempts compared to HC over a period of 2 years after suicide attempts. Similarly, Erhardt et al. (2013) have shown higher levels of CSF QUIN and higher ratio of CSF QUIN/KA in patients with suicide attempts compared to HC, on the other hand, the CSF QUIN levels in the patients have significantly decreased from the time of suicide attempts to the 6-month follow-up. Brundin et al. (2016) have reported lower levels of plasma and CSF PIC, lower ratio of plasma and CSF PIC/QUIN, and higher ratio of CSF KYN/TRP in patients with suicide attempts compared to HC, and the reductions of CSF PIC have been sustained over a period of 2 years after suicide attempts. The above-mentioned results of longitudinal follow-up studies in suicidal patients suggest sustained dysregulation of the tryptophan-kynurenine pathway possibly *via* microglial activation for a certain period after suicide attempts.

Hughes et al. (2000) examined the effects of acute tryptophan depletion on mood and suicidal ideation in patients with bipolar disorders. No significant changes in mood and suicidality scores were observed after acute plasma tryptophan depletion for 28 h in patients with bipolar disorders. In addition, using healthy adult volunteers, Dougherty et al. (2008) examined self-rated mood and somatic symptoms and the time-course of multiple plasma indicators of brain 5-HT synthesis after a 50-g depletion and loading as a comparison to the corresponding 100-g formulations that are typically used. They have manipulated L-tryptophan levels to temporarily decrease (depletion) or increase (loading) 5-HT synthesis. In both the 50- and 100-g depletion groups, negative self-ratings of mood and somatic symptoms were increased, and interestingly in the loading conditions, the 100-g formulation resulted in more negative reports of mood states, while the 50-g formulation did not and the 100-g formulation produced higher ratings of negative somatic symptoms than the 50-g formulation. Even though suicidal ideation was not measured in this healthy volunteers’ study, this study indicates the impact of shifting tryptophan metabolites for negative mood in humans. These studies are limited as they consider only short-term depletion, however, it is likely that the causal linkage between tryptophan, suicide and microglia will become clearer by combining these intervention studies and biomarker research using CSF and/or peripheral blood samples.

On the other hand, epidemiological surveys reveal that males have a disproportionately lower rate of suicide attempts and an excessively higher rate of completed suicides compared to females (O’Loughlin and Sherwood, 2005; World-Health-Organisation, 2014; Freeman et al., 2017). These epidemiological data indicate that there are independent biological pathways in suicide attempts and completed suicide based on sex differences. Higher impulse and aggression in males have been suggested to be linked to completed suicide (Mann et al., 2005). Interestingly, interactions between aggression and the tryptophan-kynurenine pathway have been suggested (Coccaro et al., 2016; Comai et al., 2016). Moreover, recent clinical research has shown that sex differences are observed in tryptophan metabolism in patients with anxiety disorder (Songtachalert et al., 2018). Therefore, we believe that gender differences in tryptophan-kynurenine metabolism might also exist in the process of suicide. Furthermore, sex differences have been observed in the process of microglial activation in rodents (Schwarz et al., 2012; Kato et al., 2013a; Lively et al., 2018; Berkiks et al., 2019). Future biological studies focusing on gender differences in suicide attempts and completed suicide *via* sex-related microglial activation should be investigated.

## Future Suicide Studies Based on Human Multi-Dimensional Analysis

Analysis using fresh microglia in human brains is an ideal method to clarify the roles of microglia in neuropsychiatric disorders, however, technological and ethical considerations have limited the ability to conduct research using fresh human microglia. Thus, alternative methods using non-brain tissues are warranted. Before concluding, we herein introduce two novel methods to predict microglia-related pathophysiologies using human blood samples. First, we have developed a novel technique to produce microglia-like cells from human blood monocytes by just adding two cytokines (IL-34 and GM-CSF) for 2 weeks (Ohgidani et al., 2014). Dynamic morphological and molecular-level analyses such as phagocytosis and cytokine releases are applicable using the iMG cells (Ohgidani et al., 2014, 2015), and we have recently revealed possible microglial pathophysiology in early-onset dementia (Nasu-Hakola disease), chronic pain (fibromyalgia) and mood disorder (bipolar disorder) by establishing and analyzing iMG cells from patients (Ohgidani et al., 2014, 2017a,b). The iMG cells can reveal both state- and trait- related characteristics by repeated analysis, and we hope that the iMG analysis from patients with suicidal behaviors can reveal microglial dynamic contributions to suicide-related behaviors. On the other hand, brain-derived exosomes have recently been a focus in understanding the pathophysiology of brain diseases without using brain biopsy (Yuyama et al., 2012; Goetzl et al., 2016; Hamlett et al., 2018). A novel method has been developed to predict the levels of proteins which are related to neuron-derived exosomes (NDE) by analyzing small amounts of human blood plasma using a sandwich immunoassay between anti-neuron antibody and antibodies against CD81 (an exosome marker) and against other proteins related to neuroinflammation and synaptic functions (Kawata et al., 2018). Using this method, we have recently reported that IL-34/CD81 levels were significantly higher in drug-free MDD patients (Kuwano et al., 2018a). IL-34 from neurons is known to be a crucial factor to maintain and activate microglia in the brain (Mizuno et al., 2011; Noto et al., 2014; Ohgidani et al., 2014). As such, research focusing on microglia-derived exosomes is warranted. At the present stage, interactions between the levels of these peripheral markers (using plasma/serum metabolites and iMG cells) and the levels of microglial activity in the brain has not been well elucidated, and we hope that these interactions will be clarified in future research.

In conclusion, we have introduced recent findings regarding the interaction between suicide and microglia based on postmortem, PET, CSF, and serum/plasma analysis of human subjects, which indicate microglial pathophysiology as a possible contribution factor of suicide and suicide-related behaviors. Human microglia research is not easy to conduct due to technical and ethical complexities, thus we believe that a multi-dimensional approach including novel techniques of blood sample analysis should be conducted to compensate for imitations in each method (Figure 1). Based on such methods, a novel biological interventional approach should be developed for suicide prevention in the near future.

## Ethics Statement

The present study was conducted in accordance with the Declaration of Helsinki and was approved by the Ethics Committee of the Graduate School of Medical Sciences, Kyushu University (29-624). The postmortem brain sample in the pilot analysis was obtained from the Human Brain Bank of the Department of Neuropathology of the Sainte-Anne Hospital consisting of autopsy examinations carried out at the Department of Pathology and Forensic Medicine of the Raymond Poincaré University Hospital, Garches, France.

## Author Contributions

TK, HS and MOh: contributed to the conception and design. TK and FC was responsible for protocol of the pilot pathological investigation. HS, MOh, FC, GL and TK contributed to the investigation. MOh, HS and TK contributed to the data checking, analysis, and interpretation of data. HS, MOh, NK, IT and TK wrote the drafted manuscript, and GL, MOn, DS, DK, MM and SK revised it critically for important intellectual content. All the authors provided final approval of the version to be published.

## Conflict of Interest Statement

The authors declare that the research was conducted in the absence of any commercial or financial relationships that could be construed as a potential conflict of interest.

## References

[B1] Banati R. B. Newcombe J. Gunn R. N. Cagnin A. Turkheimer F. Heppner F.. (2000). The peripheral benzodiazepine binding site in the brain in multiple sclerosis: quantitative *in vivo* imaging of microglia as a measure of disease activity. Brain 123, 2321–2337. 10.1093/brain/123.11.2321 11050032

[B2] Bay-Richter C. Linderholm K. R. Lim C. K. Samuelsson M. Traskman-Bendz L. Guillemin G. J.. (2015). A role for inflammatory metabolites as modulators of the glutamate N-methyl-D-aspartate receptor in depression and suicidality. Brain Behav. Immun. 43, 110–117. 10.1016/j.bbi.2014.07.012 25124710

[B3] Berkiks I. Garcia-Segura L. M. Nassiri A. Mesfioui A. Ouichou A. Boulbaroud S.. (2019). The sex differences of the behavior response to early life immune stimulation: microglia and astrocytes involvement. Physiol. Behav. 199, 386–394. 10.1016/j.physbeh.2018.11.037 30529512

[B4] Bian Q. Kato T. Monji A. Hashioka S. Mizoguchi Y. Horikawa H.. (2008). The effect of atypical antipsychotics, perospirone, ziprasidone and quetiapine on microglial activation induced by interferon-γ. Prog. Neuropsychopharmacol. Biol. Psychiatry 32, 42–48. 10.1016/j.pnpbp.2007.06.031 17716796

[B5] Block M. L. Hong J. S. (2005). Microglia and inflammation-mediated neurodegeneration: multiple triggers with a common mechanism. Prog. Neurobiol. 76, 77–98. 10.1016/j.pneurobio.2005.06.004 16081203

[B6] Bloomfield P. S. Selvaraj S. Veronese M. Rizzo G. Bertoldo A. Owen D. R.. (2016). Microglial activity in people at ultra high risk of psychosis and in schizophrenia: an [(11)C]PBR28 PET brain imaging study. Am. J. Psychiatry 173, 44–52. 10.1176/appi.ajp.2015.14101358 26472628 PMC4821370

[B7] Bradley K. A. Case J. A. Khan O. Ricart T. Hanna A. Alonso C. M.. (2015). The role of the kynurenine pathway in suicidality in adolescent major depressive disorder. Psychiatry Res. 227, 206–212. 10.1016/j.psychres.2015.03.031 25865484 PMC4430385

[B8] Brisch R. Steiner J. Mawrin C. Krzyzanowska M. Jankowski Z. Gos T. (2017). Microglia in the dorsal raphe nucleus plays a potential role in both suicide facilitation and prevention in affective disorders. Eur. Arch. Psychiatry Clin. Neurosci. 267, 403–415. 10.1007/s00406-017-0774-1 28229240 PMC5509773

[B9] Brundin L. Sellgren C. M. Lim C. K. Grit J. Palsson E. Landen M.. (2016). An enzyme in the kynurenine pathway that governs vulnerability to suicidal behavior by regulating excitotoxicity and neuroinflammation. Transl. Psychiatry 6:e865. 10.1038/tp.2016.133 27483383 PMC5022080

[B10] Bryleva E. Y. Brundin L. (2017a). Kynurenine pathway metabolites and suicidality. Neuropharmacology 112, 324–330. 10.1016/j.neuropharm.2016.01.034 26820800 PMC5998805

[B11] Bryleva E. Y. Brundin L. (2017b). Suicidality and activation of the kynurenine pathway of tryptophan metabolism. Curr. Top. Behav. Neurosci. 31, 269–284. 10.1007/7854_2016_5 27221623

[B12] Burguillos M. A. Deierborg T. Kavanagh E. Persson A. Hajji N. Garcia-Quintanilla A.. (2011). Caspase signalling controls microglia activation and neurotoxicity. Nature 472, 319–324. 10.1038/nature09788 21389984

[B13] Busse M. Busse S. Myint A. M. Gos T. Dobrowolny H. Müller U. J.. (2015). Decreased quinolinic acid in the hippocampus of depressive patients: evidence for local anti-inflammatory and neuroprotective responses? Eur. Arch. Psychiatry Clin. Neurosci. 265, 321–329. 10.1007/s00406-014-0562-0 25409655

[B14] Chesney E. Goodwin G. M. Fazel S. (2014). Risks of all-cause and suicide mortality in mental disorders: a meta-review. World Psychiatry 13, 153–160. 10.1002/wps.20128 24890068 PMC4102288

[B15] Clark S. M. Pocivavsek A. Nicholson J. D. Notarangelo F. M. Langenberg P. Mcmahon R. P.. (2016). Reduced kynurenine pathway metabolism and cytokine expression in the prefrontal cortex of depressed individuals. J. Psychiatry Neurosci. 41, 386–394. 10.1503/jpn.150226 27070351 PMC5082509

[B16] Coccaro E. F. Lee R. Fanning J. R. Fuchs D. Goiny M. Erhardt S.. (2016). Tryptophan, kynurenine, and kynurenine metabolites: relationship to lifetime aggression and inflammatory markers in human subjects. Psychoneuroendocrinology 71, 189–196. 10.1016/j.psyneuen.2016.04.024 27318828 PMC5744870

[B17] Comai S. Bertazzo A. Vachon J. Daigle M. Toupin J. Cote G.. (2016). Tryptophan via serotonin/kynurenine pathways abnormalities in a large cohort of aggressive inmates: markers for aggression. Prog. Neuropsychopharmacol. Biol. Psychiatry 70, 8–16. 10.1016/j.pnpbp.2016.04.012 27117820

[B18] Courtet P. Giner L. Seneque M. Guillaume S. Olie E. Ducasse D. (2016). Neuroinflammation in suicide: toward a comprehensive model. World J. Biol. Psychiatry 17, 564–586. 10.3109/15622975.2015.1054879 26223957

[B19] Dantzer R. O’Connor J. C. Lawson M. A. Kelley K. W. (2011). Inflammation-associated depression: from serotonin to kynurenine. Psychoneuroendocrinology 36, 426–436. 10.1016/j.psyneuen.2010.09.012 21041030 PMC3053088

[B20] Dibaj P. Steffens H. Nadrigny F. Neusch C. Kirchhoff F. Schomburg E. D. (2010). Long-lasting post-mortem activity of spinal microglia *in situ* in mice. J. Neurosci. Res. 88, 2431–2440. 10.1002/jnr.22402 20623536

[B21] Doorduin J. De Vries E. F. Willemsen A. T. De Groot J. C. Dierckx R. A. Klein H. C. (2009). Neuroinflammation in schizophrenia-related psychosis: a PET study. J. Nucl. Med. 50, 1801–1807. 10.2967/jnumed.109.066647 19837763

[B22] Dougherty D. M. Marsh-Richard D. M. Mathias C. W. Hood A. J. Addicott M. A. Moeller F. G.. (2008). Comparison of 50- and 100-g L -tryptophan depletion and loading formulations for altering 5-HT synthesis: pharmacokinetics, side effects and mood states. Psychopharmacology 198, 431–445. 10.1007/s00213-008-1163-2 18452034 PMC2818099

[B23] Erhardt S. Lim C. K. Linderholm K. R. Janelidze S. Lindqvist D. Samuelsson M.. (2013). Connecting inflammation with glutamate agonism in suicidality. Neuropsychopharmacology 38, 743–752. 10.1038/npp.2012.248 23299933 PMC3671988

[B24] Eyo U. Dailey M. E. (2012). Effects of oxygen-glucose deprivation on microglial mobility and viability in developing mouse hippocampal tissues. Glia 60, 1747–1760. 10.1002/glia.22394 22847985 PMC3786781

[B25] Fields R. D. (2008). White matter in learning, cognition and psychiatric disorders. Trends Neurosci. 31, 361–370. 10.1016/j.tins.2008.04.001 18538868 PMC2486416

[B26] Freeman A. Mergl R. Kohls E. Szekely A. Gusmao R. Arensman E.. (2017). A cross-national study on gender differences in suicide intent. BMC Psychiatry 17:234. 10.1186/s12888-017-1398-8 28662694 PMC5492308

[B27] Goetzl E. J. Mustapic M. Kapogiannis D. Eitan E. Lobach I. V. Goetzl L.. (2016). Cargo proteins of plasma astrocyte-derived exosomes in Alzheimer’s disease. FASEB J. 30, 3853–3859. 10.1096/fj.201600756R 27511944 PMC5067254

[B28] Gould T. D. Georgiou P. Brenner L. A. Brundin L. Can A. Courtet P.. (2017). Animal models to improve our understanding and treatment of suicidal behavior. Transl. Psychiatry 7:e1092. 10.1038/tp.2017.50 28398339 PMC5416692

[B29] Halaris A. Myint A. M. Savant V. Meresh E. Lim E. Guillemin G.. (2015). Does escitalopram reduce neurotoxicity in major depression? J. Psychiatr. Res. 66–67, 118–126. 10.1016/j.jpsychires.2015.04.026 26009299

[B30] Hamlett E. D. Ledreux A. Potter H. Chial H. J. Patterson D. Espinosa J. M.. (2018). Exosomal biomarkers in down syndrome and Alzheimer’s disease. Free Radic. Biol. Med. 114, 110–121. 10.1016/j.freeradbiomed.2017.08.028 28882786 PMC6135098

[B31] Harrison P. J. (1999). The neuropathology of schizophrenia. A critical review of the data and their interpretation. Brain 122, 593–624. 10.1093/brain/122.4.593 10219775

[B32] Hashioka S. (2011). Antidepressants and neuroinflammation: can antidepressants calm glial rage down? Mini Rev. Med. Chem. 11, 555–564. 10.2174/138955711795906888 21699486

[B33] Hashioka S. Klegeris A. Monji A. Kato T. Sawada M. Mcgeer P. L.. (2007). Antidepressants inhibit interferon-γ-induced microglial production of IL-6 and nitric oxide. Exp. Neurol. 206, 33–42. 10.1016/j.expneurol.2007.03.022 17481608

[B34] Hawton K. van Heeringen K. (2009). Suicide. Lancet 373, 1372–1381. 10.1016/S0140-6736(09)60372-X 19376453

[B35] Hinwood M. Morandini J. Day T. A. Walker F. R. (2012). Evidence that microglia mediate the neurobiological effects of chronic psychological stress on the medial prefrontal cortex. Cereb. Cortex 22, 1442–1454. 10.1093/cercor/bhr229 21878486

[B36] Holmes S. E. Hinz R. Conen S. Gregory C. J. Matthews J. C. Anton-Rodriguez J. M.. (2018). Elevated translocator protein in anterior cingulate in major depression and a role for inflammation in suicidal thinking: a positron emission tomography study. Biol. Psychiatry 83, 61–69. 10.1016/j.biopsych.2017.08.005 28939116

[B37] Horikawa H. Kato T. A. Mizoguchi Y. Monji A. Seki Y. Ohkuri T.. (2010). Inhibitory effects of SSRIs on IFN-γ induced microglial activation through the regulation of intracellular calcium. Prog. Neuropsychopharmacol. Biol. Psychiatry 34, 1306–1316. 10.1016/j.pnpbp.2010.07.015 20654672

[B38] Hughes J. H. Dunne F. Young A. H. (2000). Effects of acute tryptophan depletion on mood and suicidal ideation in bipolar patients symptomatically stable on lithium. Br. J. Psychiatry 177, 447–451. 10.1192/bjp.177.5.447 11059999

[B39] Kanchanatawan B. Sirivichayakul S. Ruxrungtham K. Carvalho A. F. Geffard M. Ormstad H.. (2018). Deficit, but not nondeficit, schizophrenia is characterized by mucosa-associated activation of the tryptophan catabolite (TRYCAT) pathway with highly specific increases in IgA responses directed to picolinic, xanthurenic, and quinolinic acid. Mol. Neurobiol. 55, 1524–1536. 10.1007/s12035-017-0417-6 28181189

[B43] Kato T. A. Hayakawa K. Monji A. Kanba S. (2013a). Missing and possible link between neuroendocrine factors, neuropsychiatric disorders, and microglia. Front. Integr. Neurosci. 7:53. 10.3389/fnint.2013.00053 23874274 PMC3711058

[B46] Kato T. A. Watabe M. Kanba S. (2013b). Neuron-glia interaction as a possible glue to translate the mind-brain gap: a novel multi-dimensional approach toward psychology and psychiatry. Front. Psychiatry 4:139. 10.3389/fpsyt.2013.00139 24155727 PMC3804762

[B48] Kato T. A. Yamauchi Y. Horikawa H. Monji A. Mizoguchi Y. Seki Y.. (2013c). Neurotransmitters, psychotropic drugs and microglia: clinical implications for psychiatry. Curr. Med. Chem. 20, 331–344. 10.2174/0929867311320030003 23157624

[B42] Kato T. A. Kanba S. (2013). Are microglia minding us? Digging up the unconscious mind-brain relationship from a neuropsychoanalytic approach. Front. Hum. Neurosci. 7:13. 10.3389/fnhum.2013.00013 23443737 PMC3580984

[B40] Kato T. Mizoguchi Y. Monji A. Horikawa H. Suzuki S. O. Seki Y.. (2008). Inhibitory effects of aripiprazole on interferon-γ-induced microglial activation via intracellular Ca^2+^ regulation *in vitro*. J. Neurochem. 106, 815–825. 10.1111/j.1471-4159.2008.05435.x 18429930

[B41] Kato T. Monji A. Hashioka S. Kanba S. (2007). Risperidone significantly inhibits interferon-γ-induced microglial activation *in vitro*. Schizophr. Res. 92, 108–115. 10.1016/j.schres.2007.01.019 17363222

[B44] Kato T. A. Monji A. Mizoguchi Y. Hashioka S. Horikawa H. Seki Y.. (2011a). Anti-inflammatory properties of antipsychotics via microglia modulations: are antipsychotics a ‘fire extinguisher’ in the brain of schizophrenia? Mini Rev. Med. Chem. 11, 565–574. 10.2174/138955711795906941 21699487

[B45] Kato T. A. Monji A. Yasukawa K. Mizoguchi Y. Horikawa H. Seki Y.. (2011b). Aripiprazole inhibits superoxide generation from phorbol-myristate-acetate (PMA)-stimulated microglia *in vitro*: implication for antioxidative psychotropic actions via microglia. Schizophr. Res. 129, 172–182. 10.1016/j.schres.2011.03.019 21497059

[B47] Kato T. A. Watabe M. Tsuboi S. Ishikawa K. Hashiya K. Monji A.. (2012). Minocycline modulates human social decision-making: possible impact of microglia on personality-oriented social behaviors. PLoS One 7:e40461. 10.1371/journal.pone.0040461 22808165 PMC3396661

[B49] Kawata K. Mitsuhashi M. Aldret R. (2018). A preliminary report on brain-derived extracellular vesicle as novel blood biomarkers for sport-related concussions. Front. Neurol. 9:239. 10.3389/fneur.2018.00239 29706930 PMC5906531

[B50] Kettenmann H. Hanisch U. K. Noda M. Verkhratsky A. (2011). Physiology of microglia. Physiol. Rev. 91, 461–553. 10.1152/physrev.00011.2010 21527731

[B200] Kreisel T. Frank M. G. Licht T. Reshef R. Ben-Menachem-Zidon O. Baratta M. V.. (2014). Dynamic microglial alterations underlie stress-induced depressive-like behavior and suppressed neurogenesis. Mol. Psychiatry 19, 699–709. 10.1038/mp.2013.155 24342992

[B51] Kuperberg G. R. Broome M. R. Mcguire P. K. David A. S. Eddy M. Ozawa F.. (2003). Regionally localized thinning of the cerebral cortex in schizophrenia. Arch. Gen. Psychiatry 60, 878–888. 10.1001/archpsyc.60.9.878 12963669

[B52] Kuwano N. Kato T. A. Mitsuhashi M. Sato-Kasai M. Shimokawa N. Hayakawa K.. (2018a). Neuron-related blood inflammatory markers as an objective evaluation tool for major depressive disorder: an exploratory pilot case-control study. J. Affect. Disord. 240, 88–98. 10.1016/j.jad.2018.07.040 30059939

[B53] Kuwano N. Kato T. A. Setoyama D. Sato-Kasai M. Shimokawa N. Hayakawa K.. (2018b). Tryptophan-kynurenine and lipid related metabolites as blood biomarkers for first-episode drug-naive patients with major depressive disorder: an exploratory pilot case-control study. J. Affect. Disord. 231, 74–82. 10.1016/j.jad.2018.01.014 29454180

[B54] Lively S. Wong R. Lam D. Schlichter L. C. (2018). Sex- and development-dependent responses of rat microglia to pro- and anti-inflammatory stimulation. Front. Cell. Neurosci. 12:433. 10.3389/fncel.2018.00433 30524242 PMC6262307

[B55] Maes M. Leonard B. E. Myint A. M. Kubera M. Verkerk R. (2011). The new ‘5-HT’ hypothesis of depression: cell-mediated immune activation induces indoleamine 2,3-dioxygenase, which leads to lower plasma tryptophan and an increased synthesis of detrimental tryptophan catabolites (TRYCATs), both of which contribute to the onset of depression. Prog. Neuropsychopharmacol. Biol. Psychiatry 35, 702–721. 10.1016/j.pnpbp.2010.12.017 21185346

[B56] Mann J. J. Apter A. Bertolote J. Beautrais A. Currier D. Haas A.. (2005). Suicide prevention strategies: a systematic review. JAMA 294, 2064–2074. 10.1001/jama.294.16.2064 16249421

[B57] Mechawar N. Savitz J. (2016). Neuropathology of mood disorders: do we see the stigmata of inflammation? Transl. Psychiatry 6:e946. 10.1038/tp.2016.212 27824355 PMC5314124

[B58] Mizuno T. Doi Y. Mizoguchi H. Jin S. Noda M. Sonobe Y.. (2011). Interleukin-34 selectively enhances the neuroprotective effects of microglia to attenuate oligomeric amyloid-β neurotoxicity. Am. J. Pathol. 179, 2016–2027. 10.1016/j.ajpath.2011.06.011 21872563 PMC3181379

[B59] Monji A. Kato T. Kanba S. (2009). Cytokines and schizophrenia: microglia hypothesis of schizophrenia. Psychiatry Clin. Neurosci. 63, 257–265. 10.1111/j.1440-1819.2009.01945.x 19579286

[B60] Monji A. Kato T. A. Mizoguchi Y. Horikawa H. Seki Y. Kasai M.. (2013). Neuroinflammation in schizophrenia especially focused on the role of microglia. Prog. Neuropsychopharmacol. Biol. Psychiatry 42, 115–121. 10.1016/j.pnpbp.2011.12.002 22192886

[B61] Müller N. Myint A. M. Krause D. Weidinger E. Schwarz M. J. (2013). Anti-inflammatory treatment in schizophrenia. Prog. Neuropsychopharmacol. Biol. Psychiatry 42, 146–153. 10.1016/j.pnpbp.2012.11.008 23178230

[B62] Myint A. M. (2012). Kynurenines: from the perspective of major psychiatric disorders. FEBS J. 279, 1375–1385. 10.1111/j.1742-4658.2012.08551.x 22404766

[B63] Myint A. M. Schwarz M. J. Müller N. (2012). The role of the kynurenine metabolism in major depression. J. Neural Transm. 119, 245–251. 10.1007/s00702-011-0741-3 22139324

[B64] Nakagami Y. Kubo H. Katsuki R. Sakai T. Sugihara G. Naito C.. (2018). Development of a 2-h suicide prevention program for medical staff including nurses and medical residents: a two-center pilot trial. J. Affect. Disord. 225, 569–576. 10.1016/j.jad.2017.08.074 28886497

[B65] Ni R. Mu L. Ametamey S. (2018). Positron emission tomography of type 2 cannabinoid receptors for detecting inflammation in the central nervous system. Acta Pharmacol. Sin. [Epub ahead of print]. 10.1038/s41401-018-0035-5 29921889 PMC6460366

[B66] Nobuhara K. Okugawa G. Sugimoto T. Minami T. Tamagaki C. Takase K.. (2006). Frontal white matter anisotropy and symptom severity of late-life depression: a magnetic resonance diffusion tensor imaging study. J. Neurol. Neurosurg. Psychiatry 77, 120–122. 10.1136/jnnp.2004.055129 16361611 PMC2117392

[B67] Noto D. Sakuma H. Takahashi K. Saika R. Saga R. Yamada M.. (2014). Development of a culture system to induce microglia-like cells from haematopoietic cells. Neuropathol. Appl. Neurobiol. 40, 697–713. 10.1111/nan.12086 24016036 PMC4282385

[B68] Ogyu K. Kubo K. Noda Y. Iwata Y. Tsugawa S. Omura Y.. (2018). Kynurenine pathway in depression: a systematic review and meta-analysis. Neurosci. Biobehav. Rev. 90, 16–25. 10.1016/j.neubiorev.2018.03.023 29608993

[B69] Ohgidani M. Kato T. A. Kanba S. (2015). Introducing directly induced microglia-like (iMG) cells from fresh human monocytes: a novel translational research tool for psychiatric disorders. Front. Cell. Neurosci. 9:184. 10.3389/fncel.2015.00184 26074765 PMC4444822

[B70] Ohgidani M. Kato T. A. Haraguchi Y. Matsushima T. Mizoguchi Y. Murakawa-Hirachi T.. (2017a). Microglial CD206 gene has potential as a state marker of bipolar disorder. Front. Immunol. 7:676. 10.3389/fimmu.2016.00676 28119691 PMC5220016

[B71] Ohgidani M. Kato T. A. Hosoi M. Tsuda M. Hayakawa K. Hayaki C.. (2017b). Fibromyalgia and microglial TNF-α: translational research using human blood induced microglia-like cells. Sci. Rep. 7:11882. 10.1038/s41598-017-11506-4 28928366 PMC5605512

[B72] Ohgidani M. Kato T. A. Sagata N. Hayakawa K. Shimokawa N. Sato-Kasai M.. (2016). TNF-α from hippocampal microglia induces working memory deficits by acute stress in mice. Brain Behav. Immun. 55, 17–24. 10.1016/j.bbi.2015.08.022 26551431

[B73] Ohgidani M. Kato T. A. Setoyama D. Sagata N. Hashimoto R. Shigenobu K.. (2014). Direct induction of ramified microglia-like cells from human monocytes: dynamic microglial dysfunction in Nasu-Hakola disease. Sci. Rep. 4:4957. 10.1038/srep04957 24825127 PMC4019954

[B74] O’Loughlin S. Sherwood J. (2005). A 20-year review of trends in deliberate self-harm in a British town, 1981–2000. Soc. Psychiatry Psychiatr. Epidemiol. 40, 446–453. 10.1007/s00127-005-0912-3 16003594

[B75] Pandey G. N. Rizavi H. S. Ren X. Fareed J. Hoppensteadt D. A. Roberts R. C.. (2012). Proinflammatory cytokines in the prefrontal cortex of teenage suicide victims. J. Psychiatr. Res. 46, 57–63. 10.1016/j.jpsychires.2011.08.006 21906753 PMC3224201

[B76] Pandey G. N. Rizavi H. S. Zhang H. Bhaumik R. Ren X. (2018). Abnormal protein and mRNA expression of inflammatory cytokines in the prefrontal cortex of depressed individuals who died by suicide. J. Psychiatry Neurosci. 43:170192. 10.1503/jpn.170192 30371993 PMC6203549

[B77] Penzes P. Cahill M. E. Jones K. A. Vanleeuwen J. E. Woolfrey K. M. (2011). Dendritic spine pathology in neuropsychiatric disorders. Nat. Neurosci. 14, 285–293. 10.1038/nn.2741 21346746 PMC3530413

[B78] Rubenowitz E. Waern M. Wilhelmson K. Allebeck P. (2001). Life events and psychosocial factors in elderly suicides—a case-control study. Psychol. Med. 31, 1193–1202. 10.1017/s0033291701004457 11681545

[B79] Rupprecht R. Papadopoulos V. Rammes G. Baghai T. C. Fan J. Akula N.. (2010). Translocator protein (18 kDa) (TSPO) as a therapeutic target for neurological and psychiatric disorders. Nat. Rev. Drug Discov. 9, 971–988. 10.1038/nrd3295 21119734

[B80] Sato-Kasai M. Kato T. A. Ohgidani M. Mizoguchi Y. Sagata N. Inamine S.. (2016). Aripiprazole inhibits polyI:C-induced microglial activation possibly via TRPM7. Schizophr. Res. 178, 35–43. 10.1016/j.schres.2016.08.022 27614570

[B81] Schnieder T. P. Trencevska I. Rosoklija G. Stankov A. Mann J. J. Smiley J.. (2014). Microglia of prefrontal white matter in suicide. J. Neuropath. Exp. Neur. 73, 880–890. 10.1097/nen.0000000000000107 25101704 PMC4141011

[B82] Schwarz J. M. Sholar P. W. Bilbo S. D. (2012). Sex differences in microglial colonization of the developing rat brain. J. Neurochem. 120, 948–963. 10.1111/j.1471-4159.2011.07630.x 22182318 PMC3296888

[B83] Setiawan E. Attwells S. Wilson A. A. Mizrahi R. Rusjan P. M. Miler L.. (2018). Association of translocator protein total distribution volume with duration of untreated major depressive disorder: a cross-sectional study. Lancet Psychiatry 5, 339–347. 10.1016/s2215-0366(18)30048-8 29496589

[B84] Setiawan E. Wilson A. A. Mizrahi R. Rusjan P. M. Miler L. Rajkowska G.. (2015). Role of translocator protein density, a marker of neuroinflammation, in the brain during major depressive episodes. JAMA Psychiatry 72, 268–275. 10.1001/jamapsychiatry.2014.2427 25629589 PMC4836849

[B85] Setoyama D. Kato T. A. Hashimoto R. Kunugi H. Hattori K. Hayakawa K.. (2016). Plasma metabolites predict severity of depression and suicidal ideation in psychiatric patients-a multicenter pilot analysis. PLoS One 11:e0165267. 10.1371/journal.pone.0165267 27984586 PMC5161310

[B86] Sierra A. de Castro F. Del Rio-Hortega J. Iglesias-Rozas J. R. Garrosa M. Kettenmann H. (2016). The “big-bang” for modern glial biology: translation and comments on pio del rio-hortega 1919 series of papers on microglia. Glia 64, 1801–1840. 10.1002/glia.23046 27634048

[B87] Songtachalert T. Roomruangwong C. Carvalho A. F. Bourin M. Maes M. (2018). Anxiety disorders: sex differences in serotonin and tryptophan metabolism. Curr. Top. Med. Chem. 18, 1704–1715. 10.2174/1568026618666181115093136 30430940

[B88] Steiner J. Bielau H. Brisch R. Danos P. Ullrich O. Mawrin C.. (2008). Immunological aspects in the neurobiology of suicide: elevated microglial density in schizophrenia and depression is associated with suicide. J. Psychiatr. Res. 42, 151–157. 10.1016/j.jpsychires.2006.10.013 17174336

[B89] Steiner J. Mawrin C. Ziegeler A. Bielau H. Ullrich O. Bernstein H. G.. (2006). Distribution of HLA-DR-positive microglia in schizophrenia reflects impaired cerebral lateralization. Acta Neuropathol. 112, 305–316. 10.1007/s00401-006-0090-8 16783554

[B90] Steiner J. Walter M. Gos T. Guillemin G. J. Bernstein H. G. Sarnyai Z.. (2011). Severe depression is associated with increased microglial quinolinic acid in subregions of the anterior cingulate gyrus: evidence for an immune-modulated glutamatergic neurotransmission? J. Neuroinflammation 8:94. 10.1186/1742-2094-8-94 21831269 PMC3177898

[B91] Su F. Yi H. Xu L. Zhang Z. (2015). Fluoxetine and S-citalopram inhibit M1 activation and promote M2 activation of microglia *in vitro*. Neuroscience 294, 60–68. 10.1016/j.neuroscience.2015.02.028 25711936

[B92] Sublette M. E. Galfalvy H. C. Fuchs D. Lapidus M. Grunebaum M. F. Oquendo M. A.. (2011). Plasma kynurenine levels are elevated in suicide attempters with major depressive disorder. Brain Behav. Immun. 25, 1272–1278. 10.1016/j.bbi.2011.05.002 21605657 PMC3468945

[B93] Sudol K. Mann J. J. (2017). Biomarkers of suicide attempt behavior: towards a biological model of risk. Curr. Psychiatry Rep. 19:31. 10.1007/s11920-017-0781-y 28470485

[B94] Sugama S. Fujita M. Hashimoto M. Conti B. (2007). Stress induced morphological microglial activation in the rodent brain: involvement of interleukin-18. Neuroscience 146, 1388–1399. 10.1016/j.neuroscience.2007.02.043 17433555

[B95] Suzuki K. Sugihara G. Ouchi Y. Nakamura K. Futatsubashi M. Takebayashi K.. (2013). Microglial activation in young adults with autism spectrum disorder. JAMA Psychiatry 70, 49–58. 10.1001/jamapsychiatry.2013.272 23404112

[B96] Takano A. Arakawa R. Ito H. Tateno A. Takahashi H. Matsumoto R.. (2010). Peripheral benzodiazepine receptors in patients with chronic schizophrenia: a PET study with [^11^C]DAA1106. Int. J. Neuropsychopharmacol. 13, 943–950. 10.1017/s1461145710000313 20350336

[B97] Thornicroft G. Sartorius N. (1993). The course and outcome of depression in different cultures: 10-year follow-up of the WHO collaborative study on the assessment of depressive disorders. Psychol. Med. 23, 1023–1032. 10.1017/s0033291700026489 8134505

[B98] Torres-Platas S. G. Comeau S. Rachalski A. Bo G. D. Cruceanu C. Turecki G.. (2014a). Morphometric characterization of microglial phenotypes in human cerebral cortex. J. Neuroinflammation 11:12. 10.1186/1742-2094-11-12 24447857 PMC3906907

[B99] Torres-Platas S. G. Cruceanu C. Chen G. G. Turecki G. Mechawar N. (2014b). Evidence for increased microglial priming and macrophage recruitment in the dorsal anterior cingulate white matter of depressed suicides. Brain Behav. Immun. 42, 50–59. 10.1016/j.bbi.2014.05.007 24858659

[B100] Tynan R. J. Naicker S. Hinwood M. Nalivaiko E. Buller K. M. Pow D. V.. (2010). Chronic stress alters the density and morphology of microglia in a subset of stress-responsive brain regions. Brain Behav. Immun. 24, 1058–1068. 10.1016/j.bbi.2010.02.001 20153418

[B101] van Berckel B. N. Bossong M. G. Boellaard R. Kloet R. Schuitemaker A. Caspers E.. (2008). Microglia activation in recent-onset schizophrenia: a quantitative (*R*)-[^11^C]PK11195 positron emission tomography study. Biol. Psychiatry 64, 820–822. 10.1016/j.biopsych.2008.04.025 18534557

[B102] Watabe M. Kato T. A. Tsuboi S. Ishikawa K. Hashiya K. Monji A.. (2013). Minocycline, a microglial inhibitor, reduces ‘honey trap’ risk in human economic exchange. Sci. Rep. 3:1685. 10.1038/srep01685 23595250 PMC3629414

[B103] WHO (2018). Suicide Data [Online]. Available online at: http://www.who.int/mental_health/prevention/suicide/suicideprevent/en/. [Accessed on 9 July 2018].

[B104] World-Health-Organisation (2014). Preventing Suicide. A Global Imperative. Geneva: World Health Organisation.

[B105] Yuyama K. Sun H. Mitsutake S. Igarashi Y. (2012). Sphingolipid-modulated exosome secretion promotes clearance of amyloid-β by microglia. J. Biol. Chem. 287, 10977–10989. 10.1074/jbc.m111.324616 22303002 PMC3322859

