# Supplementary material for: Suicide and Microglia: Recent Findings and Future Perspectives Based on Human Studies
Source: Front Cell Neurosci. 2019 Feb 13;13:31. doi: 10.3389/fncel.2019.00031 (PMC6381042; doi:10.3389/fncel.2019.00031)
Supplement: Supplementary file 3 [file Table_3.docx]

**Supplement document**

**Subjects and Methods**

1. **Sampling collection**

The postmortem brain sample in the pilot analysis was obtained from the Human Brain Bank of the Department of Neuropathology of the Sainte-Anne Hospital consisting of autopsy examinations carried out at the Department of Pathology and Forensic Medicine of the Raymond Poincaré University Hospital, Garches, France. The brain was removed during a general autopsy and then fixed in a 10% formalin solution for at least 3 weeks before the neuropathological examination. This pilot neuropathological analysis was conducted in the Neuropathology Department of the Sainte-Anne Hospital and in the Human Histopathology and Animal Models Laboratory of the Institute Pasteur, Paris, France.

**2. 3D morphological analysis of microglia in a suicide victim.**

The brain block (frontal cortex) was cut into 100μm-thick sections using Vibratome (LeicaMicrosystems, Germany). Sections were blocked for 24 hours with 3% bovine serum albumin (BSA) in PBS containing 1% Triton X-100 and 0.1% formalin at 4 ℃. After blocking, the sections were incubated with rabbit polyclonal anti-Iba-1 antibody (Wako, Japan) for 4 days at 4 ℃. After washing with PBS, the sections were incubated with a mixture of Alexa488-conjugated goat anti-rabbit IgG antibody (Invitrogen, USA) overnight at 4 ℃. The sections were counterstained with DAPI (4',6'diamino-2-phenylindole) and mounted with Fluoromount G (interchim, France). Fluorescent images were acquired with spinning disc confocal laser scanning microscope (Cell Voyager CV1000, Yokogawa, Japan). The 3D images of microglial cells were created with Imaris 7.0 software (Bitplane, Zurich, Switzerland). Compared to 2D analysis, 3D reconstruction techniques enable us to measure more detailed morphological parameters of a single microglial cell such as volume and surface area. For example, the volume and surface area of the whole cell body of the microglial cell shown in **Figure 1** (upper right: green) were 989 μm^3^ and 1884 μm^2^, respectively. The volume and surface area of the cell somata of the cell in **Figure 1** (upper right: red) were 295 μm^3^ and 262 μm^2^, respectively.
